# Granulocyte Colony-Stimulating Factor Combined with Methylprednisolone Improves Functional Outcomes in Rats with Experimental Acute Spinal Cord Injury

**DOI:** 10.6061/clinics/2018/e235

**Published:** 2018-02-12

**Authors:** William Gemio Jacobsen Teixeira, Alexandre Fogaça Cristante, Raphael Martus Marcon, Gustavo Bispo, Ricardo Ferreira, Tarcísio Eloy Pessoa de Barros-Filho

**Affiliations:** I Divisao de Cirurgia da Coluna, Tumores da Coluna, Instituto do Cancer do Estado de Sao Paulo (ICESP), Hospital das Clinicas HCFMUSP, Faculdade de Medicina, Universidade de Sao Paulo, Sao Paulo, SP, BR; II Divisao de Cirurgia da Coluna, Laboratorio de Investigacao Medica, Instituto de Ortopedia e Traumatologia (IOT), Hospital das Clinicas HCFMUSP, Faculdade de Medicina, Universidade de Sao Paulo, Sao Paulo, SP, BR; III Laboratorio de Investigacao Medica – 41 (LIM-41), Instituto de Ortopedia e Traumatologia (IOT), Hospital das Clinicas HCFMUSP, Faculdade de Medicina, Universidade de Sao Paulo, Sao Paulo, SP, BR

**Keywords:** Spinal Cord Injuries, Wistar Rats, Granulocyte Colony-Stimulating Factor, Methylprednisolone

## Abstract

**OBJECTIVES:**

To evaluate the effects of combined treatment with granulocyte colony-stimulating factor (G-CSF) and methylprednisolone in rats subjected to experimental spinal cord injury.

**METHODS:**

Forty Wistar rats received a moderate spinal cord injury and were divided into four groups: control (no treatment); G-CSF (G-CSF at the time of injury and daily over the next five days); methylprednisolone (methylprednisolone for 24 h); and G-CSF/Methylprednisolone (methylprednisolone for 24 h and G-CSF at the time of injury and daily over the next five days). Functional evaluation was performed using the Basso, Beattie and Bresnahan score on days 2, 7, 14, 21, 28, 35 and 42 following injury. Motor-evoked potentials were evaluated. Histological examination of the spinal cord lesion was performed immediately after euthanasia on day 42.

**RESULTS:**

Eight animals were excluded (2 from each group) due to infection, a normal Basso, Beattie and Bresnahan score at their first evaluation, or autophagy, and 32 were evaluated. The combination of methylprednisolone and G-CSF promoted greater functional improvement than methylprednisolone or G-CSF alone (*p*<0.001). This combination also exhibited a synergistic effect, with improvements in hyperemia and cellular infiltration at the injury site (*p*<0.001). The groups displayed no neurophysiological differences (latency *p*=0.85; amplitude *p*=0.75).

**CONCLUSION:**

Methylprednisolone plus G-CSF promotes functional and histological improvements superior to those achieved by either of these drugs alone when treating spinal cord contusion injuries in rats. Combining the two drugs did have a synergistic effect.

## INTRODUCTION

Researchers worldwide struggle to find evidence of substances or procedures that can reduce the devastating consequences of spinal cord injuries, which affect up to 80 per million people worldwide, with 500,000 new cases per year 1. Most research is focused on surgical treatment, rehabilitation or physical training and particularly on pharmacological therapies aimed at restoring neuronal plasticity and function by reducing the effects of secondary lesions and stimulating tissue regeneration 2-5. Estrogen has been found to reduce pro-inflammatory activity and protect neurons 6,7. The antioxidant effect of melatonin has also been extensively investigated, but only in low-quality experimental studies 8. Methylprednisolone has proven clinically effective 9,10; however, its use as a first-line treatment has recently been questioned due to the high incidence of complications and adverse events, especially when higher doses are used 11. The use of methylprednisolone has thus been reevaluated 12 but is still widely accepted 13. Stem-cell transplantation 14,15 has also been investigated.

Granulocyte colony-stimulating factor (G-CSF) is a glycoprotein that acts as a growth-stimulating factor for hematopoietic progenitor cells 16,17. G-CSF is used to treat neutropenia and to mobilize hematopoietic stem cells in cases of bone marrow transplantation 16. Studies show that G-CSF also has non-hematopoietic functions and that it can increase tissue regeneration in organs such as the brain and heart 17,18.

G-CSF has also been shown to produce neurological improvements in experimental models of spinal cord injury 17,19-21. In the acute phase of injury, G-CSF mobilized bone marrow cells to the injured spinal cord, where it suppressed neuronal and oligodendrocyte apoptosis, the expression of inflammatory cytokines such as tumor necrosis factor-alpha (TNF-α) and interleukin-1 beta (IL-1 beta) 22, and lipid peroxidation 23, in addition to stimulating the production of neurotrophic factors 21. In the subacute phase of traumatic spinal cord injury, G-CSF stimulated angiogenesis 24 and suppressed glial scar formation 25. In two phase I/IIa studies, some neurological recovery was observed in most patients with spinal cord injuries 26,27.

It is also possible that some drugs may act synergistically to alleviate spinal cord injury 28, and synergy between pharmacological and physical factors may improve trauma recovery 2,29. Indeed, the best strategy in situations with a complex pathophysiology, as in the case of spinal cord injury, likely involves treatments that employ different pathogenic mechanisms 29. However, no study has yet investigated the effects of combining methylprednisolone and G-CSF, which can be investigated using an experimental model of spinal cord injury in rats.

Therefore, our objective was to evaluate the effects of G-CSF associated with methylprednisolone on functional, neurophysiological and histological outcomes in a standardized experimental rat model of spinal cord injury.

## MATERIALS AND METHODS

### Ethics

This experimental study was carried out in strict accordance with all international guidelines on handling and controlling pain or suffering in the care and use of animals in research. According to our laboratory protocols, all animals are handled and stimulated to move prior to the experiments so that they can become accustomed to the researchers and to the motor function evaluation procedures. *Ad libitum* feeding and hydration are maintained during studies carried out in the lab, and appropriate cages (40 x 60 cm) and housing are provided, with a maximum of five animals per cage in a space with controlled temperature and humidity. The animals were submitted to surgical and experimental procedures and were euthanized under general anesthesia.

The study protocol was evaluated and approved by the Research Ethics Committee of the institution (Comissão Científica do Instituto de Ortopedia e Traumatologia e pela Comissão de Ética para Análise de Projetos de Pesquisa do Hospital das Clínicas da Faculdade de Medicina da Universidade de São Paulo (CAPPesq – HC-FMUSP; permit number: IOT no. 965).

### Study design, sample size and experimental animals

This is a controlled study with Wistar rats, which were divided into four groups. Sample size was based on previously published studies using approximately 10 animals per group 9,29. In total, 40 male Wistar rats, aged 20 to 21 weeks old and initially weighing 300 to 340 g, were used. All animals came from the same university vivarium and were weighed at the beginning and at the end of the study. All rats were clinically evaluated at baseline to ensure that they had normal motricity and good health. Animals with macroscopic evidence of abnormalities in the spinal cord or functional problems were excluded. Animals that died shortly after the experimental spinal cord lesion, or animals with autophagy or mutilation behaviors, were also excluded from the study. Normal motricity (21 points on the Basso, Beattie and Bresnahan (BBB) scale), even after the experimental spinal cord lesion, was also an exclusion criterion, since normal functioning indicated that the procedure failed.

The 40 animals were randomly allocated (using sequence generator software and a ratio of 1:1:1:1) into five groups, with 10 animals per group. The Control Group (CG) received no treatment at all after the experimental spinal cord injury. The Methylprednisolone Group (MG) was treated with 30 mg/kg of intravenous methylprednisolone at 10 min, 6 h and 24 h after the spinal cord lesion, according to the protocol described by Park et al. 30. The G-CSF group received treatment with 15 mcg/kg of subcutaneous G-CSF after the lesion was induced and then daily for 5 days, according to the method described by Kawabe et al. 24. Finally, the G-CSF-M group received G-CSF at the moment the experimental lesion was induced, followed by daily treatment with G-CSF for 5 days, as well as methylprednisolone 24 h after the lesion was induced. G-CSF and methylprednisolone were provided at 10 min, 6 h and 24 h after the spinal cord lesions were given to this group, at the same doses used for the other two groups

### Procedures

All animals were operated on by a single surgeon. The animals were anesthetized using 50 mg/kg of pentobarbital administered intraperitoneally. The anesthetic effect was expected to begin within five minutes and to last approximately two hours, which was long enough for the spinal cord injury procedure to be carried out. The level of anesthesia was determined by evaluating the withdrawal reflex to tail compression. After spinal cord injury, the animals were treated with oral tramadol (15 mg/kg) dissolved in water 31.

All animals underwent a laminectomy under anesthesia. A moderate contusion spinal cord lesion was produced at T10, as described previously 29, using an NYU-Impactor device with a 10-g impact rod from a 12.5 mm-height to compress the spinal cord for 15 s. This procedure produced a spinal cord lesion that created loss of locomotor function.

After surgery and spinal cord lesion, the animals were transferred to chambers with controlled temperature (25°C to 28°C), where they remained for 30 min. The bladders were emptied by external compression. Antibiotic prophylaxis was performed with cephalothin administered subcutaneously (25 mg/kg) immediately after the lesion and once a day for seven subsequent days. All rats were kept in the same vivarium and under the same controlled environmental conditions for 42 days before being euthanized by a lethal dose of pentobarbital (140 mg/kg).

The spinal cord was removed for necropsy. A segment from T8 to T12 (approximately 2.5 cm-long) was fixed and prepared for histological analysis as described previously 29. Axial slices were cut 2 mm apart to span the injured area and were extended 1 cm proximally and caudally from the center of the lesion. After fixation and production of paraffin blocks, 5-μm-thick sections were produced and stained with hematoxylin-eosin for histological evaluation.

### Outcomes evaluation

The pathologist responsible for the histological analysis (not one of the authors), who was blinded to the experimental groups, scored the sections for the presence of necrosis, hemorrhage, hyperemia, neural tissue degeneration, and infiltrate as follows: 0 (absent), 1 (mild), 2 (moderate) and 3 (extensive).

Function was evaluated using the BBB rating scale 29,32,33. This 21-point scale ranges from complete paraplegia (score 0) to normal neurological function (score 21) and was applied for 4 to 5 min on days 2, 7, 14, 21, 28, 35, and 42 after spinal cord lesioning. The BBB score on the 42nd day after injury was assessed simultaneously by two trained observers who were blinded to experimental conditions and to each other's evaluations. In cases of disagreement, the lower score was recorded for analysis.

On the 42^nd^ day after lesioning, anesthetized rats (pentobarbital, 50 mg/kg) underwent a motor evoked potential (MEP) exam according to the method of Letaif et al. 6, which was performed by a researcher who was blinded to the experimental conditions, with electrodes positioned in the semitendinosus and the biceps muscles of the thigh. Amplitude and latency data were recorded.

### Statistical analysis

Data are presented as means and standard deviations in the descriptive analysis. The primary outcome of this study was the BBB score on the 42^nd^ day after injury. The histological variables and MEP results were secondary outcomes.

For inferential statistics, the data behaviors were observed for possible outliers. Once identified, outliers were submitted to the Dixon Q test for possible rejection. Subsequently, the data were subjected to the Kolmogorov-Smirnov normality test. If the data showed a normal distribution, parametric tests, e.g., Student’s t-test and analysis of variance (ANOVA), were performed. The number of factors was adjusted for each situation. Parametric analysis was also performed when the distribution was asymmetric. When the asymmetry was to the right, the data were transformed by logarithm to the base of 10, and when it was to the left, the data were raised to the square.

The BBB scores were compared using a mixed effects model with two factors, four groups and six weeks of evaluation, while considering repeated measures over time. A possible effect of interaction between these factors was also evaluated. The mixed effects model was adjusted by considering matrices of equal covariance in the different groups, as well as in unstructured form.

For the MEP results, the distribution of amplitude and latency data was initially compared between the four groups using the Kruskal-Wallis test. Next, ANOVA was used to compare the amplitude and latency means in different groups. The histological variables were also compared between groups using the Kruskal-Wallis test.

We started with a null hypothesis considering a 5% probability of type I error. Statistical analyses were performed using the Statistical Package for Social Sciences (SPSS), version 19.0zx.

## RESULTS

A total of 40 animals were operated on, but two rats had to be excluded from each group. In the CG, one rat was excluded due to failure of the experimental lesion (i.e., the rat showed normal function after the procedure), and the second was excluded for autophagy on the 22^nd^ postoperative day. In the MG group, animals were excluded due to infection on the 8^th^ and 10th postoperative days. In the G-CSF group, one animal was excluded due to lesion failure (i.e., normal BBB) and the other due to infection on the 5^th^ postoperative day. Finally, in the G-CSF-M group, animals were excluded due to lesion failure and autophagy (on the 10^th^ postoperative day).

The final sample consisted of eight animals in each group and therefore 16 limbs. Because the BBB scores in both limbs of the same animal were considered equivalent, Table 1 shows the data on the BBB evaluation for 16 cases per group at seven evaluation times. From the 14^th^ day on, the BBB scores differed significantly among the groups. The highest score was obtained in the G-CSF-M group, with a difference of 1.44 points on the 14^th^ day and 5.44 on the 42^nd^ day from the score in the CG (untreated). Multiple (post-hoc) comparisons are shown in Table 2 and confirm the superior recovery of the G-CSF-M group.

Because the MEP results were also equivalent in the forelimbs and hindlimbs, this evaluation was performed for 16 individuals per group. The MEP results in the forelimbs did not differ significantly, either for latency (*p*=0.09) or amplitude values (*p*=0.20), and the hindlimbs also showed no significant differences (*p*=0.85 and 0.08 respectively; Table 3).

**Figure 1:**
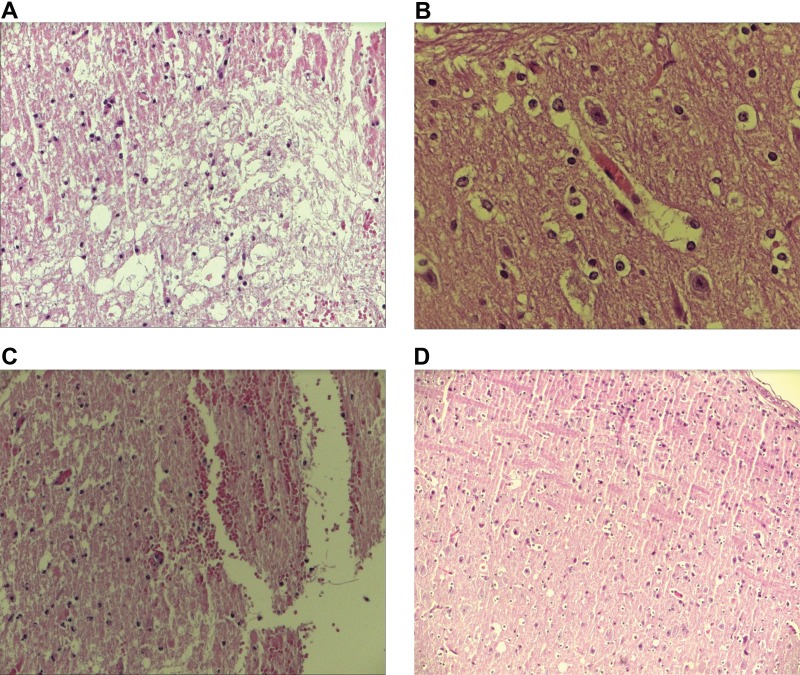
Examples of hematoxylin-eosin-stained histological slides showing the area of the spinal cord lesion. A - intense degeneration of neural tissue (200x magnification); B - intense hyperemia (400x magnification); C - moderate degree of infiltration and cystic degeneration (100x magnification) and D - an area close to the spinal cord lesion with no alterations (100x magnification).

Table 4 shows the results of the histological analysis. No significant difference was observed for necrosis, but the other histological variables were significantly different among the groups. The multiple comparison test (Mann-Whitney) showed significant differences for hyperemia, hemorrhage, degeneration of neural tissue and infiltration, as shown in Table 5. Figure 1 shows examples of histological evaluations and an example of a normal spinal cord.

## DISCUSSION

One of the reasons why traumatic spinal cord injury is such a devastating condition is that it leads to chronic impairment and disability: it impairs the function of young accident victims in the productive phase of their lives, and with the aging of the population, it is also increasingly affecting the elderly 1,34. Despite numerous advances in diagnostic technology, rehabilitation and surgical decompression therapies, which have reduced morbidity and mortality, improvements in the therapeutic approach to secondary lesions are still mostly experimental. No treatment has yet to provide a cure or to facilitate complete functional recovery for patients with spinal cord injury. In the present study, we explored whether a combination of substances will have a better effect on functional and histological recovery than one isolated drug, possibly due to synergistic effects or to the fact that different substances may act in different pathways as part of a complex physiopathological process. This hypothesis proved to be accurate: the combination of G-CSF and methylprednisolone proved to be better than the control treatment and either treatment alone, according to the functional evaluations from the second week of treatment in rats. The functional results were also better than those observed in other studies using G-CSF alone 20,31.

Primary spinal cord injury develops into a secondary injury via several biological processes, resulting in the impairment of viable tissues and leading to necrosis and neuronal apoptosis 16. The goal of pharmacological therapy is to reduce or avoid secondary injury by inhibiting inflammation, lipid peroxidation and excitotoxicity 35.

Several drugs have been studied in experimental and clinical trials 2,9. Many drugs showed promising results in experimental animal studies, but few have proven effective in humans. Of these, methylprednisolone, tirilazad mesylate, naloxone, and GM-1 ganglioside were evaluated in human clinical trials in patients with spinal cord injury 36.

Methylprednisolone was the most extensively studied drug 10,36. Although its mechanism of action is not fully known, methylprednisolone has been shown to stabilize membranes and preserve the hematospinal cord barrier, potentially reducing vasogenic edema. It also increases spinal cord blood flow, changes the electrolyte concentrations at the injury site, inhibits endorphin release, reduces free radical availability, and reduces the inflammatory response 11,36. Despite its worldwide acceptance in the treatment of spinal cord injury, methodological problems in clinical trials and new evidence from clinical studies and literature reviews have raised concerns about the small effect sizes of the clinical benefits, as well as the risks of complications associated with the use of methylprednisolone 36. Therefore, new studies should be conducted with promising drugs, including but not limited to methylprednisolone, tirilazad mesylate, naloxone and GM-1 36. G-CSF is also a promising drug in this field, as interesting effects have been shown in clinical studies of humans with spinal cord injury and thoracic myelopathy 26,27.

We therefore decided to investigate G-CSF and methylprednisolone together, compared with no therapy or with either drug alone, in the treatment of rats with spinal cord injury. We used a standardized experimental model that is widely used in this field, which produced a contusion lesion similar to the trauma most frequently seen in humans 34. Because the sensitivity of other functional analyses is still being evaluated 8, we used the BBB locomotor function scale, a validated, reproducible and widely used tool for assessing the recovery of rats after experimental spinal cord injury 29,32. We also took care to ensure that the evaluators performing the histological, functional and MEP analyses were blinded to the experimental conditions and to each other's evaluations. Due to these standardized procedures, the results can be compared with those of other studies.

The effect size of the improvement on the BBB locomotor function scale for the G-CSF group was similar to that found in other studies using the same methodology 20,31. Similar to Kadota et al. 20, we used a dose of 15 mcg/kg/day of G-CSF for five days. Dittgen et al. 31 used a higher dose comprising a single 60 mcg bolus followed by 30 mg/kg/day through an infusion pump. Considering that the functional results of all these studies were similar, the benefit of the drug can likely be achieved at lower doses and with a shorter administration than that reported by Dittgen et al. 31.

In the present study, we found no difference between groups in the MEP exam. MEP was chosen to evaluate the function of the corticospinal tract 6. The absence of significant differences in MEP in this study may be due to the death of two animals per group, which reduced the size of the final sample.

In the histological analysis, groups that received G-CSF, with or without methylprednisolone, showed improved hyperemia. All experimental groups had better results for hemorrhage and degeneration than the control group. The groups that received methylprednisolone, with or without G-CSF, showed improvements in cellular infiltration. When methylprednisolone was combined with G-CSF, all parameters improved, suggesting a synergistic effect between the two drugs on the reduction of inflammation and maintenance of tissue architecture. These findings reinforce the importance of combining different treatment modalities or drugs for diseases with complex physiopathology, such as spinal cord injuries.

Therefore, in clinical settings, different drugs with synergistic effects can be administered at smaller doses when combined, thereby improving efficacy and reducing side effects. G-CSF has already been studied in humans in phase I/IIa trials, with satisfactory results in the treatment of spinal cord injury 26,27 and thoracic myelopathy. G-CSF should be studied in phase III clinical trials. Further experimental studies are needed to evaluate the efficacy and mechanism of action underlying the synergistic effect of G-CSF and methylprednisolone. The present study is somewhat limited by the small sample size in each group. We were also unable to address adverse effects or complications due to experimental intervention.

The combination of methylprednisolone and G-CSF in the treatment of experimental spinal cord contusion injury in rats resulted in functional improvement, as assessed by the BBB scale, that was superior to the improvement obtained with either methylprednisolone or G-CSF alone. The association of methylprednisolone and G-CSF also had a synergistic effect that resulted in an improvement in hyperemia and cellular infiltration at the lesion site.

## AUTHOR CONTRIBUTIONS

Teixeira WG conceived and designed the experiments and methodology, performed the experiments, with data curation, formal analysis and investigations, analyzed the data, contributed to the writing and editing of the manuscript, contributed to project and resources/funding administration. Cristante AF conceived and designed the experiments and methodology, analyzed the data, contributed to the writing and editing of the manuscript, contributed to project and resources/funding administration. Marcon RM conceived and designed the experiments and methodology, performed the experiments, with data curation, formal analysis and investigations, analyzed the data. Bispo G and Ferreira R performed the experiments, with data curation, formal analysis and investigations. Barros-Filho TE conceived and designed the experiments and methodology, analyzed the data, contributed to the writing and editing of the manuscript, contributed to project and resources/funding administration.

## Figures and Tables

**Table 1:** Basso, Beattie, and Bresnahan (BBB) rating scores for hindlimbs, according to the evaluation times after spinal cord injury: mean, minimum (min) and maximum (max) values, standard deviations (SD), degrees of freedom (DF) and results of the chi-squared and Kruskal-Wallis tests.

Day	Group	N	Mean	SD	Min	Max	*χ^2^*	DF	*p*
2	CG	16	0.31	0.60	0.0	2.0	0.905	3	0.824
	MG	16	0.31	0.60	0.0	2.0			
	G-CSF	16	0.50	0.73	0.0	2.0			
	G-CSF-M	16	0.44	0.73	0.0	2.0			
	CG	16	1.69	2.89	0.0	9.0			
7	MG	16	1.13	0.62	0.0	3.0	4.938	3	0.176
	G-CSF	16	0.63	0.89	0.0	2.0			
	G-CSF-M	16	1.00	1.71	0.0	5.0			
14	CG	16	4.94	2.11	1.0	8.0	16.634	3	**0.001**
	MG	16	4.38	2.33	1.0	9.0			
	G-CSF	16	3.13	1.59	1.0	5.0			
	G-CSF-M	16	6.38	1.78	3.0	8.0			
21	CG	16	6.06	2.69	2.0	11.0	16.126	3	**0.001**
	MG	16	7.63	3.32	1.0	14.0			
	G-CSF	16	5.44	1.71	2.0	8.0			
	G-CSF-M	16	8.81	2.14	5.0	12.0			
28	CG	16	6.88	2.39	4.0	11.0	21.408	3	**<0.001**
	MG	16	9.56	1.82	7.0	12.0			
	G-CSF	16	7.88	1.36	5.0	10.0			
	G-CSF-M	16	11.38	3.46	5.0	15.0			
35	CG	16	7.56	2.78	5.0	12.0	21.204	3	**<0.001**
	MG	16	10.13	1.63	8.0	12.0			
	G-CSF	16	9.31	1.62	8.0	12.0			
	G-CSF-M	16	13.19	3.37	8.0	18.0			
42	CG	16	8.69	2.09	6.0	12.0	21.644	3	**<0.001**
	MG	16	11.00	1.83	8.0	14.0			
	G-CSF	16	10.38	1.59	8.0	12.0			
	G-CSF-M	16	14.13	3.59	8.0	18.0			

CG = control group; MG = methylprednisolone group; G-CSF = granulocyte colony-stimulating factor group and G-CSF-M = group receiving G-CSF and methylprednisolone.

**Table 2:** Multiple comparisons of BBB scores for limbs according to the evaluation times from the second week after spinal cord injury.

Day	Reference group	Comparison group	*p*
14	CG	CG	1
		MG	0.37
		G-CSF	0.02
		G-CSF-M	0.07
	MG	CG	0.37
		MG	1
		G-CSF	0.17
		G-CSF-M	0.01
	G-CSF	CG	0.02
		MG	0.17
		G-CSF	1
		G-CSF-M	<0.001
	G-CSF-M	CG	0.07
		MG	0.01
		G-CSF	<0.001
		G-CSF-M	1
21	CG	CG	1
		MG	0.2
		G-CSF	0.51
		G-CSF-M	0.01
	MG	CG	0.2
		MG	1
		G-CSF	0.02
		G-CSF-M	0.14
	G-CSF	CG	0.51
		MG	0.02
		G-CSF	1
		G-CSF-M	<0.001
	G-CSF-M	CG	0.01
		MG	0.14
		G-CSF	<0.001
		G-CSF-M	1
28	CG	CG	1
		MG	<0.001
		G-CSF	0.14
		G-CSF-M	<0.001
	MG	CG	<0.001
		MG	1
		G-CSF	0.02
		G-CSF-M	0.05
	G-CSF	CG	0.14
		MG	0.02
		G-CSF	1
		G-CSF-M	<0.001
	G-CSF-M	CG	<0.001
		MG	0.05
		G-CSF	0
		G-CSF-M	1
35	CG	CG	1
		MG	0.01
		G-CSF	0.04
		G-CSF-M	<0.001
	MG	CG	0.01
		MG	1
		G-CSF	0.13
		G-CSF-M	0.01
	G-CSF	CG	0.04
		MG	0.13
		G-CSF	1
		G-CSF-M	<0.001
	G-CSF-M	CG	<0.001
		MG	0.01
		G-CSF	<0.001
		G-CSF-M	1
42	CG	CG	1
		MG	<0.001
		G-CSF	0.02
		G-CSF-M	<0.001
	MG	CG	<0.001
		MG	1
		G-CSF	0.27
		G-CSF-M	0.01
	G-CSF	CG	0.02
		MG	0.27
		G-CSF	1
		G-CSF-M	<0.001
	G-CSF-M	CG	<0.001
		MG	0.01
		G-CSF	<0.001
		G-CSF-M	1

CG = control group; MG = methylprednisolone group; G-CSF = granulocyte colony-stimulating factor group and G-CSF-M = group receiving G-CSF and methylprednisolone.

**Table 3:** Latency and amplitude values in the motor-evoked potential exam (means, standard deviations, minimum and maximum values) of hindlimbs of rats subjected to experimental spinal cord lesion and comparisons between groups using the chi-squared and Kruskal-Wallis tests.

Hindlimbs	Groups	N (limbs)	Mean (ms)	SD	Min	Max	*χ^2^*	DF	*p*
**Latency**	CG	16	27.83	8.77	15.80	40.30	6.626	3	0.85
	MG	16	23.71	3.74	16.30	29.10			
	G-CSF	16	22.36	7.30	12.50	34.20			
	G-CSF-M	16	21.30	8.20	12.40	40.30			
**Amplitude**	CG	16	26.86	14.78	6.00	50.10	6.898	3	0.075
	MG	16	43.98	65.22	4.70	276.80			
	G-CSF	16	64.71	92.85	10.90	312.10			
	G-CSF-M	16	97.43	115.58	4.70	483.00			

CG = control group; MG = methylprednisolone group; G-CSF = granulocyte colony-stimulating factor group and G-CSF-M = group receiving G-CSF and methylprednisolone.

**Table 4:** Histological analysis scores according to the variables necrosis, hemorrhage, hyperemia, degeneration, and cellular infiltrate: mean, minimum (min) and maximum (max) values, standard deviations (SD), degrees of freedom (DF) and chi-squared and Mann-Whitney test results.

Variable	Group	N	Mean score (0-3)	SD	Min	Max	*χ^2^*	DF	*p*
Necrosis	CG	8	1.33	0.87	0	3	6.858	3	0.077
	MG	8	0.92	0.5	0	2			
	G-CSF	8	1.04	0.46	0	2			
	G-CSF-M	8	0.83	0.48	0	2			
Hemorrhage	CG	8	1.54	0.72	1	3	14.23	3	**0.003**
	MG	8	1.08	0.65	0	2			
	G-CSF	8	0.96	0.55	0	2			
	G-CSF-M	8	0.83	0.48	0	2			
Hyperemia	CG	8	1.83	0.64	1	3	22.51	3	**<0.001**
	MG	8	1.38	0.65	0	2			
	G-CSF	8	1.25	0.61	1	3			
	G-CSF-M	8	0.96	0.46	0	2			
Degeneration	CG	8	1.92	0.65	1	3	16.9	3	**0.001**
	MG	8	1.42	0.65	0	2			
	G-CSF	8	1.29	0.62	0	2			
	G-CSF-M	8	1.08	0.65	0	2			
Cellular infiltrate	CG	8	1.92	0.78	1	3	19.01	3	**<0.001**
	MG	8	1.29	0.81	0	3			
	G-CSF	8	1.54	0.59	1	3			
	G-CSF-M	8	0.92	0.65	0	2			

CG = control group; MG = methylprednisolone group; G-CSF = granulocyte colony-stimulating factor group and G-CSF-M = group receiving G-CSF and methylprednisolone.

**Table 5:** Multiple comparisons between groups in relation to histological evaluation (Mann-Whitney test).

Groups	Comparison	Group	*p*
Hyperemia	CG	CG	1.00
		MG	0.608
		G-CSF	**0.002**
		G-CSF-M	**<0.001**
	MG	CG	0.608
		MG	1.000
		G-CSF	0.311
		G-CSF-M	**0.011**
	G-CSF	CG	**0.002**
		MG	0.311
		G-CSF	1.00
		G-CSF-M	0.077
	G-CSF-M	CG	**<0.001**
		MG	**0.011**
		G-CSF	0.077
		G-CSF-M	1.000
Hemorrhage	CG	CG	1.00
		MG	**0.047**
		G-CSF	**0.005**
		G-CSF-M	**<0.001**
	MG	CG	**0.047**
		MG	1.000
		G-CSF	0.466
		G-CSF-M	0.144
	G-CSF	CG	**0.005**
		MG	0.466
		G-CSF	1.00
		G-CSF-M	0.419
	G-CSF-M	CG	**<0.001**
		MG	0.144
		G-CSF	0.419
		G-CSF-M	1.000
Degeneration	CG	CG	1.00
		MG	**0.019**
		G-CSF	**0.003**
		G-CSF-M	**<0.001**
	MG	CG	**0.019**
		MG	1.000
		G-CSF	0.448
		G-CSF-M	**0.076**
	G-CSF	CG	**0.003**
		MG	0.448
		G-CSF	1.00
		G-CSF-M	0.265
	G-CSF-M	CG	**<0.001**
		MG	0.076
		G-CSF	0.265
		G-CSF-M	1.000
Cellular infiltration	CG	CG	1.00
		MG	**0.016**
		G-CSF	0.088
		G-CSF-M	**<0.001**
	MG	CG	**0.016**
		MG	1.00
		G-CSF	0.288
		G-CSF-M	0.089
	G-CSF	CG	0.088
		MG	0.288
		G-CSF	1.00
		G-CSF-M	**0.002**
	G-CSF-M	CG	**<0.001**
		MG	0.089
		G-CSF	**0.002**
		G-CSF-M	1.000

CG = control group; MG = methylprednisolone group; G-CSF = granulocyte colony-stimulating factor group and G-CSF-M = group receiving G-CSF and methylprednisolone.
